# Child and Adolescent Mental Health Service (CAMHS) in Poland—From the Perspective of the Current State and New Reform

**DOI:** 10.3390/healthcare13162078

**Published:** 2025-08-21

**Authors:** Monika Serkowska, Marlena Robakowska, Dariusz Aleksander Rystwej, Michał Brzeziński

**Affiliations:** 1 Department of Paediatrics, Gastroenterology, Allergology & Paediatric Nutrition, Medical University of Gdansk, 80-210 Gdansk, Poland; brzezinski@gumed.edu.pl; 2 Division of Public Health & Social Medicine, Department of Social Medicine, Medical University of Gdansk, 80-210 Gdansk, Poland; marlena.robakowska@gumed.edu.pl; 3 Student Scientific Circle Interdisciplinary Health Care Management, Department of Public Health & Social Medicine and 2nd Division of Radiology, Medical University of Gdansk, 80-210 Gdansk, Poland; d.rystwej@gumed.edu.pl

**Keywords:** CAMHS, mental health, children, adolescents, health care

## Abstract

Introduction: The organization of mental health care is undergoing a transformation from an institutionalized model to a community-centered model. Due to the critical specialist workforce shortage, insufficient funding, and the large number of children in crisis, its implementation presents a challenge. The aim of this study is to analyze the current situation regarding access to system-based care under contracts with the National Health Fund in various provinces in Poland. Materials and Methods: Based on an analysis of data, resources available to patients were assessed—specifically, information was obtained from the National Health Fund website entitled “NFZ Treatment Waiting Times.” From this, the waiting times for appointments in child and adolescent mental health care facilities, the availability of mental health care facilities under contracts with the National Health Fund in Poland, legal acts, and data from the Central Statistical Office were extracted. Then, an analysis of the current accessibility to child and adolescent mental health services was conducted. The inclusion criteria for data sources were as follows: accessibility—the data had to be openly available to researchers without restrictions; credibility—the data had to be verified by individual health care facilities; usefulness—the data had to accurately reflect the actual availability of services and the needs within the child and adolescent psychiatric care system. Results: There are significant differences and deviations from the average number of facilities and waiting times when comparing the 16 provinces. Notably, some of the analyzed facilities are already operating within the framework of Child and Adolescent Mental Health Centers, where the mean waiting period for inpatient care is 105 days, the mean waiting period for day-care units is 61 days, and the mean waiting period for outpatient clinics is 257 days. The number of facilities is increasing under the reform, with new level I reference centers being opened, which ensures prevention and early support is provided by a pedagogue, psychologist, and non-medical staff, providing enhanced accessibility to care without the need for a visit to a child and adolescent psychiatrist, of whom there are only 579 for the entire child population in Poland. This metric primarily refers to first-time appointments in public institutions, with notable disparities between urban and rural areas. Conclusions: The development of the reform offers hope for quicker access to mental health support for children and adolescents. With the consistent implementation of the reform and further support from non-governmental organizations, there is a high chance of building an effective community-based model with a short waiting time for help and reducing ineffective hospitalizations, among other things, in terms of costs.

## 1. Introduction

The health care system in Poland is based on universal health insurance, administered and financed by the National Health Fund (NFZ). The NFZ funds healthcare services from public resources, collected primarily through mandatory health insurance contributions. The Ministry of Health serves as the governing body overseeing national health policy, developing strategies and programs, and supervising the activities of the NFZ. The system operates within three main segments of service provision: primary healthcare, ambulatory specialist care, and hospital treatment [1]. The organization of mental health services is regulated by legal provisions and the guidelines of health programs, including the National Mental Health Protection Programme [2,3].

Child and adolescent mental health care is a topic of discussion in many countries, even on a global scale, though not with the same intensity in every community [4]. In Poland, it is also a frequent topic of discussion regarding support for children’s and adolescents’ health, as children with mental health issues are still not adequately cared for [5,6,7,8]. This topic is particularly important because it is estimated that over half a million children in Poland suffer from mental disorders, with over 200,000 aged 7–11 years and over 350,000 aged 12–17 years [2]. Due to significant staffing shortages (including only 579 child and adolescent psychiatrists) and insufficient funding, amounting to just 3.5% of the National Health Fund’s (known as in Poland as the “NFZ”—“Narodowy Fundusz Zdrowia”) budget for all mental health care and addiction treatment services for children and adults, necessary ward closures and outpatient clinic shutdowns have become common. As a result, the current state of psychiatric health care organization is viewed as very difficult, if not critical [2,9].

The previous (old) model of child and adolescent psychiatric care was characterized by a significant number of individuals waiting for services, the predominance of inpatient treatment, and the absence of a preventive component, which limited the effectiveness of interventions and led to fragmented care. The new model, currently being implemented as part of the reform, is structured around three reference levels, introducing a community-based approach, elements of prevention and early intervention, an expanded network of support facilities, and uniform organizational standards. Although implementation is still ongoing and waiting lists for services persist, the new model enhances the comprehensiveness and coordination of care, shifting the focus from isolated, costly inpatient treatment to integrated community-based support, optimizing both financial and care-related outcomes [2].

In this article, the term community-based mental health care model refers to an organized system of psychiatric care in which most diagnostic, therapeutic, and rehabilitative activities are delivered within the patient’s natural living environment—their local community—rather than in isolated hospital facilities. This model assumes the decentralization of services, the development of a network of first-contact facilities (e.g., mental health centers, outpatient clinics, mobile teams), and the integration of psychiatric care with other forms of support, including social, educational, and legal assistance.

It is important to distinguish between community-based and community-centered. A community-based model defines the structure and location of services—they are embedded in the community and accessible close to the patient’s place of residence. A community-centered model, on the other hand, emphasizes the active involvement of the community and the patient in the planning, decision-making, and evaluation of care, giving them the role of co-creators of services. In practice, both approaches may complement each other; however, in the case of the child and adolescent psychiatry reform in Poland, the primary foundation is community-based, with the gradual implementation of community-centered elements.

The fundamental assumptions of international programs (MH action plans) include increasing the role and actions of mental health promotion and the prevention of mental disorders, creating and supporting mental health care systems and organizations, conducting scientific research in this area, and implementing strategies for development and advocacy [10,11]. This largely aligns with the actions being taken in Poland and with other action strategies, such as the WHO Mental Health Action Plan and the National Mental Health Programs.

Like many other countries—with other examples including Germany—Poland is facing the consequences of the COVID-19 pandemic, which not only exposed but also exacerbated problems in mental health care due to isolation, anxiety caused by the threat, and a lack of security and stability. This has prompted policy-makers to initiate telepsychiatry projects [12]. Germany and Poland both face issues with access to care and the need for its integration and support at all levels [13,14]. The pandemic significantly exacerbated mental health challenges, increasing demand for services while exposing systemic weaknesses.

In line with current actions outlined, among others, in the National Mental Health Programs (the current one covering the years of 2023–2030), a reform is being implemented that is seen as a hope for improving the current situation. Newly established facilities are striving to quickly shift the mental health care model towards a community-based approach [15]. The existing institutionalized model is quite outdated, grounded in the belief that psychiatric patients should be isolated. This approach not only fosters stigmatization but also leads to fragmented care. Furthermore, since it relies on inpatient hospitalization, it is highly costly due to the expenses and length of stays, rehospitalizations, and the lack of continuity in care. Such situations can not only hinder patients’ health improvement but also exacerbate disabilities or even increase feelings of helplessness. The community-centered model, on the other hand, offers a completely different approach, aiming for comprehensive and coordinated treatment in the patient’s place of residence, as early as possible and with minimal invasiveness. In this model, care coordination is also linked with social assistance—legal, social, and other forms of help for individuals in a mental health crisis. Also important here is the position of the patient in the system, where in addition to the staff, he or she is also a decision-maker and has a say in the treatment.

Currently, there are two institutional mental health care systems operating within the public sector. The first, which existed before the reform, is characterized by a fragmented structure due to the lack of or minimal connection between units within the mental health care system—outpatient clinics, day units, and inpatient wards.

It is worth mentioning that no referral is required to see a psychiatrist, but a referral is needed to access other services (such as psychotherapy, hospitalization in a day unit or inpatient care in a planned mode). Referrals can be issued by a psychiatrist or a primary care physician.

A new solution is the introduction of Child and Adolescent Mental Health Centers, which are gradually being expanded [16,17]. These centers offer facilities, with 3 levels of service. Level I comprises centers that employ psychologists, psychotherapists, and community therapists. The services offered include psychological counseling, both individual and group psychotherapy sessions, and community-based interventions such as home visits [18].

The next level of care is the so-called level II reference centers. These take two forms. The first type consists of purely outpatient clinics, while the second combines outpatient services with day wards, providing more comprehensive care and contributing to the establishment of new day units. The staff at these centers include psychiatrists, psychologists, and psychotherapists, and in centers with day units, occupational therapists are also employed. No referral is required to access services at level I and II centers. The most advanced and intervention-focused structure consists of level III reference centers. Although they employ the same specialists as the Child and Adolescent Mental Health Centers (level II reference centers with wards), these facilities offer highly specialized, round-the-clock mental health care. Due to their inpatient nature, a referral is required for admission to such centers, except in emergency life-threatening situations. All information about the reform, as well as the requirements and best practices, is provided in the substantive and organizational standards for each reference level outlined in the reform [2,19,20,21,22].

### Aim

The aim of this study is to analyze the current systemic solutions, providing mental health care services for children and adolescents in Poland under the National Health Fund. The analysis will focus on the accessibility of facilities and the rules for utilizing them in relation to the child population, both in the context of the “old system” and the child and adolescent psychiatry reform model, with a comparison to solutions in other OECD countries. Additionally, a comparison with Central and Eastern European countries, which share Poland’s historical and economic background, would provide further insights into region-specific challenges and solutions, which might be valuable in the future.

## 2. Materials and Methods

Information regarding the functioning and organization of mental health care for children and adolescents in Poland was analyzed based on legal regulations such as the Mental Health Act [23] and the National Mental Health Programs [2,24]. The material includes data from the National Health Fund databases regarding locations providing mental health care services [17,25], for the years 2023–2024, collected between December 2023 and February 2024, as well as data from the Central Statistical Office concerning the child and adolescent population in Poland [26].

We analyzed the gathered data on the accessibility of mental health care facilities to the populations of various provinces in Poland, and at the national level, in terms of the number of facilities and waiting times. The number of facilities was compared to the number of children per facility, both for facilities within the pilot program and those not covered by the reform. Due to the types of facilities analyzed, the analysis took into account clinics, day wards, inpatient wards and new support facilities for children and youth at the first reference level. Regarding waiting times, the average time was calculated. The applied evaluation approach was based on three key criteria: usefulness, effectiveness, and relevance. Each criterion was further elaborated through guiding questions designed to enable both qualitative and quantitative assessments of the program. Specifically, the evaluation focused on the following: usefulness—the extent to which the program addresses the needs of children and adolescents, and whether it effectively resolves the problems they face; effectiveness—the degree to which the program’s objectives were achieved, taking into account the planned timeline, budget, and quality of service delivery; relevance—the alignment of program activities with actual mental health challenges and initial program assumptions, including a comparative analysis of different stages of the pilot implementation.

The results were compared with solutions in OECD countries (Germany, Norway, and the United States) based on an analysis of articles from Google Scholar, PubMed, Web of Science, and Scopus databases, using keywords such as “CAMHS,” “Child and adolescent,” “mental health,” and “healthcare.” The selection of publications was based on their topical relevance, scientific credibility, and recency. Priority was given to peer-reviewed articles, governmental documents, and official reports, addressing child and adolescent mental health care systems.

Additionally, the actions related to the WHO Mental Health Action Plan and the National Mental Health Program were verified in terms of the current international and local situation and progress in their implementation. Additionally, a comparison with Central and Eastern European countries, which share Poland’s historical and economic background, would provide further insights into region-specific challenges and solutions. The comparison was made due to the common global challenges in the mental health of children and adolescents. As the study did not involve the direct participation of human subjects, approval from a bioethics committee was not required.

## 3. Results

In Poland, between December 2023 and early January 2024, there were 212 outpatient clinics, 80 day-care units, and 41 inpatient wards for children and adolescents. Although the national average number of inpatient wards per province is 3, the national average number of day-care units is 5, and the national average number of outpatient clinics is around 14, there are considerable inequalities in nearly every province, also called a voivodeship.

**Figure 1:**
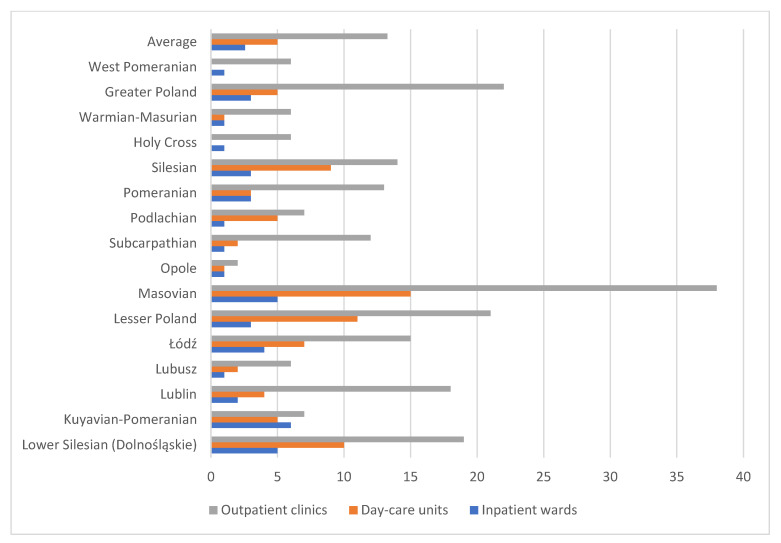
Number of facilities by type of care in various Polish provinces.

In the Figure 1 is particularly noticeable shortage of day-care units exists in two provinces: Holy Cross (Świętokrzyskie) and West Pomeranian (Zachodniopomorskie). The highest number of facilities is in the Masovian (Mazowieckie) province (58), which includes Poland’s capital, Warsaw. However, when analyzing the maximum number of facilities by type, the Masovian province has the highest number of outpatient clinics (38), nearly three times the national average, as well as the highest number of day-care units (15), In terms of inpatient wards, the Kuyavian-Pomeranian (Kujawsko-Pomorskie) province has the most, with 6 facilities, twice the national average. On the other hand, looking at the lowest numbers, aside from the mentioned lack of day-care units, the Opole (Opolskie) province has the fewest outpatient clinics (2), which is seven times below the average. Several provinces have only one inpatient ward each—Lubusz (Lubuskie), Opole (Opolskie), Subcarpathian (Podkarpackie), Podlachia (Podlaskie), Holy Cross (Świętokrzyskie), Warmian-Masurian (Warmińsko-Mazurskie), and West Pomeranian (Zachodniopomorskie)—representing nearly half of the country. Overall, the Opole province has the fewest facilities providing mental health care for children and adolescents, with only 4 facilities. This highlights a significant disparity in the number of mental health care facilities across the country when viewed territorially by province.

**Figure 2:**
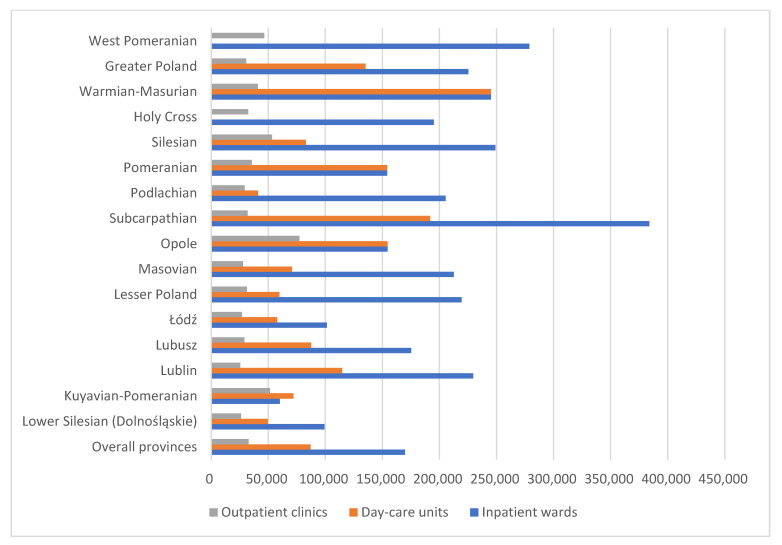
Number of children per facility in various Polish provinces.

The below diagram (Figure 2) shows the number of children per facility of a specific type in each province. The division includes outpatient clinics, inpatient wards, and day-care units. This is a much more accurate measure of needs than the raw number of facilities in a given province. The average number of children per facility is as follows: nearly 33,000 children per outpatient clinic, just over 87,000 children per day-care unit, and almost 170,000 potential patients per inpatient ward. Looking at the Figure 2 the demand for facilities is most evident in the Subcarpathian (Podkarpackie) and Warmian-Masurian (Warmińsko-Mazurskie) provinces. However, when analyzing the number of children per facility, besides the previously mentioned lack of day-care units in two provinces, the highest number of children per inpatient ward is in the Subcarpathian (Podkarpackie) province (nearly 39,000), while the highest number of children per outpatient clinic is in the Opole (Opolskie) province (just over 77,000). The lowest number of potential patients per facility is just over 26,000 in the Lower Silesian (Dolnośląskie) province for outpatient clinics, over 41,000 in the Podlachian (Podlaskie) province for day-care units, and more than 64,000 children per inpatient ward in the Kuyavian-Pomeranian (Kujawsko-Pomorskie) province. This demonstrates that the number of facilities in a given province significantly differs from the number of patients per facility.

**Figure 3:**
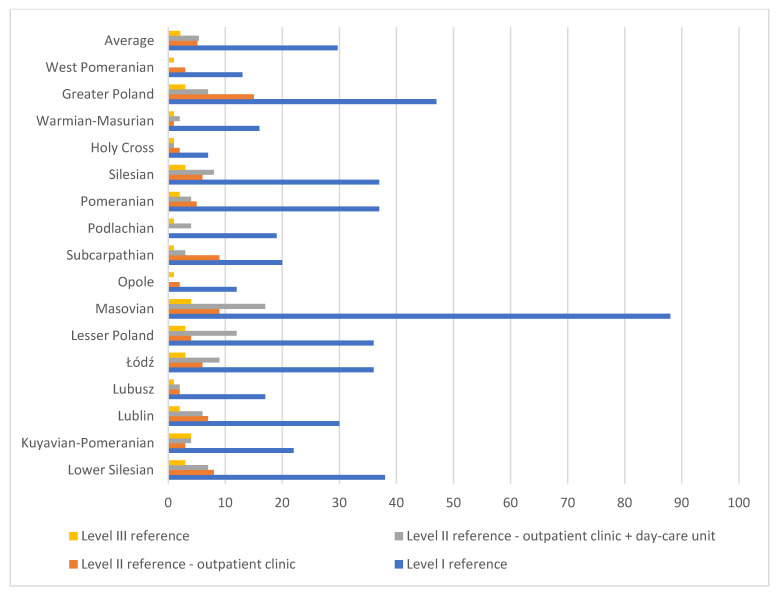
Number of facilities by reference levels in various Polish provinces.

Figure 3 shows, that currently, there are 677 facilities operating under the new reform in Poland, including 82 at reference level I, 82 at reference level II functioning exclusively as outpatient clinics, 86 at reference level II combining outpatient clinics and day-care units, and 34 at reference level III. As shown in Table 1, the Masovian (Mazowieckie) province has the highest number of facilities. However, there are significant differences compared to the number of entities in the so-called “old system” for several reasons. In this case, unlike previous data, reference level I outpatient clinics, which do not provide strictly psychiatric services, are included, and there are many of them. This helps provide support to individuals in acute crisis. Additionally, for higher levels, particularly the level II, there is a difference in the structure of facilities (outpatient clinic + day-care unit), which ensures more comprehensive care. As a result, aside from the Masovian (Mazowieckie) province, the provinces of Greater Poland (Wielkopolskie), Silesian (Śląskie), Lesser Poland (Małopolskie), Łódź (Łódzkie), and Lower Silesian (Dolnośląskie) are also well-equipped with pilot facilities. There is also a noticeable predominance of reference level I centers, which provide significant support in these provinces and partially offset the differences in the number of facilities, making half of Poland above the average in terms of the number of centers available for people in crisis. Unfortunately, there are still areas where these centers are very few. This is particularly evident in the Opole (Opolskie) province (16), where the number of facilities does not even reach 50% of the average, and in the Holy Cross (Świętokrzyskie) province (11), where the number is less than one-third of the average facility availability (45).

**Figure 4:**
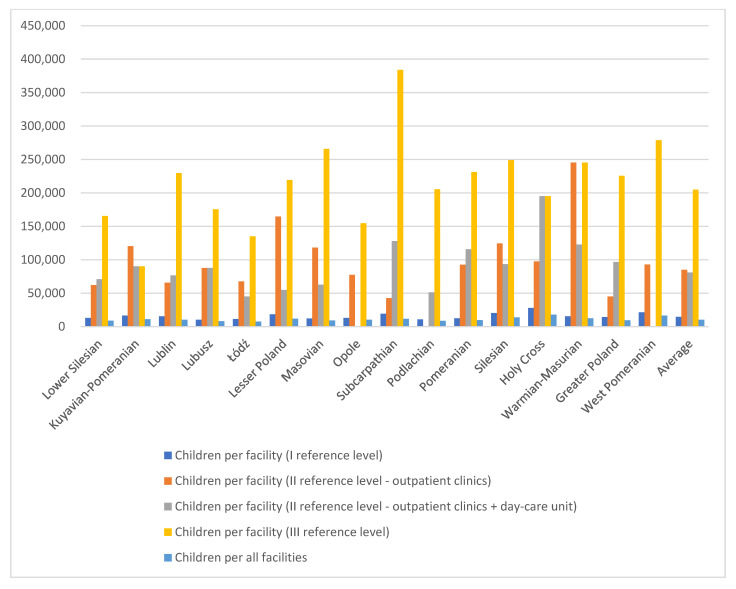
Number of children per facility by reference levels across various Polish provinces.

The below Figure 4 shows that level I reference facilities are currently the most accessible within the pilot program. The greater the number of facilities in a given region and the smaller the number of children, the greater the availability of services. This is primarily due to the fact that their establishment began much earlier, and their operation did not require the employment of child and adolescent psychiatrists. Level III reference facilities started to emerge over the past year, utilizing existing resources in the form of inpatient wards. It is noticeable, however, that there are no level II entities with the outpatient clinic + day-care unit structure in the West Pomeranian (Zachodniopomorskie) and Opole (Opolskie) provinces.

It is worth noting that outpatient clinics, day-care units, and inpatient wards are listed in the service appointment search engine funded by the National Health Fund [25,27]. This allows for an analysis of service availability based on the waiting times for services in the aforementioned entities, as shown in the table below.

**Figure 5:**
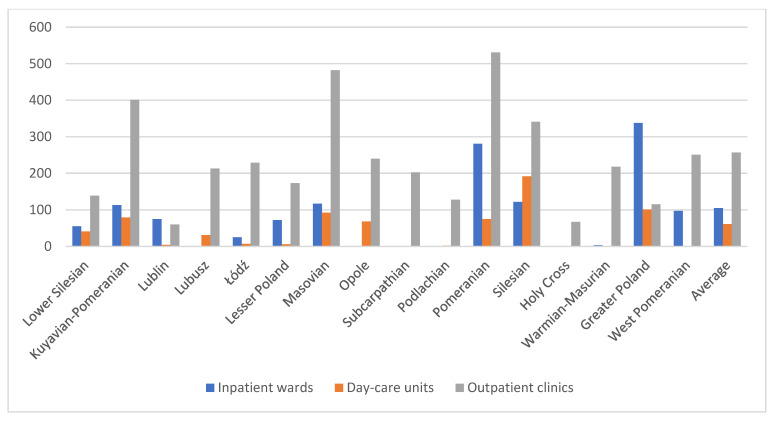
Average waiting time for services (days) in various Polish provinces.

The Figure 5 shows the mean waiting period, broken down by outpatient clinics, day-care units, and inpatient wards, under the “old system”. The longest waiting times are in the Greater Poland (Wielkopolskie), Pomeranian (Pomorskie), and Silesian (Śląskie) provinces, with 338, 281, and 122 days, respectively. This is due to the ongoing lack of beds in wards, as patients are frequently admitted on an emergency basis, such as after a suicide attempt. The Figure 5 highlights the mean waiting period for an appointment at a child and adolescent mental health outpatient clinic, which is often the first point of contact within the systemic mental health care. The fact that no referral is required to visit the clinic has little significance here, as the mean waiting period is 257 days, with the shortest being 60 days in the Lublin (Lubelskie) province and the longest being 531 days in the Pomeranian (Pomorskie) province, although this is an average. The longest waiting time is in one of the facilities in the Masovian (Mazowieckie) province, where it is 1718 days. Given that a psychiatrist diagnoses and prescribes treatment while also recommending the type of therapy (with psychotherapy in the system having a waiting time of several years), even in the early stages of a crisis, this reaction time is far too long. This metric refers primarily to first-time appointments in public institutions, with notable disparities between urban and rural areas.

**Table 1:** Comparison of evaluation criteria for the previous and current models of child and adolescent mental health care in Poland.

Criterion	Evaluation Questions	Assessment/Answer	
		Old	New
Usefulness	Does the model meet the needs of the target population?	No—the model is characterized by prolonged waiting times for the initial diagnostic appointment and the commencement of therapy, which limits its accessibility and effectiveness. Furthermore, in its current form, it does not incorporate a preventive component, which is crucial from the perspective of early intervention and preventing the escalation of mental health problems.	Yes—it provides a preventive and early intervention approach.
	Does the model address the actual problems faced by children and adolescents?	No—prolonged waiting times for services and the predominance of an isolation-based model focused primarily on inpatient treatment limit the effectiveness of interventions, contributing to the deterioration of mental health among children and adolescents.	Partially—although waiting lists for services persist, the network of support facilities has been expanded and preventive measures have been implemented, which partially improves the accessibility and comprehensiveness of care.
Effectiveness	Have the objectives of the model been achieved?	None—the model does not define precise operational objectives or priority intervention areas, limiting itself to the general assumption of the need to provide psychiatric care for the child and adolescent population.	Partially—the model is in the process of implementation. The network of facilities at all levels is currently being expanded—level I units are being established, and level II and III facilities are being further developed; new entities are being created, and existing ones are undergoing restructuring.
	Was the model implemented within the planned timeframe?	Not applicable—the model represents a potentially permanent solution aimed at meeting fundamental societal needs. Only the development and implementation of a new model, surpassing the current one in usefulness, could be evaluated in terms of completion within a defined timeframe.	No—implementation has been ongoing continuously since 2020. No specific completion date has been set.
	Was the model implemented within the planned budget?	No—the system is characterized by chronic underfunding. Although the annual Financial Plan of the National Health Fund specifies the scope of funding for psychiatric services, the available resources remain insufficient to fully achieve the model’s objectives.	No—the funds allocated for the pilot program are insufficient. Furthermore, during the COVID-19 pandemic, contracts were signed for teleconsultations and in-person visits; if the required proportion of in-person consultations was not met, facilities faced financial penalties, which in some cases forced them to close.
	Was the model implemented while maintaining the specified quality indicators?	None—no uniform quality indicators have been defined in the Polish system, which results from the lack of an annual, comprehensive evaluation enabling the formulation of objectives for the subsequent reporting period. The system relies on a reactive approach focused on ad hoc problem-solving, neglecting prevention and long-term planning.	Quality indicators have been defined in the organizational standards for facilities—top-down organizational guidelines applicable throughout the entire model have been developed [14,15].
Adequacy	Does the model address the current and actual mental health problems?	No—there is a high incidence rate combined with insufficient system resources.	Yes—comprehensive actions are planned, including prevention, hospitalization, and community-based interventions.
	Is the model consistent with the initial assumptions?	Yes—the earlier model was based on an organizational structure adapted from other medical specialties, encompassing both outpatient clinics and inpatient wards.	Yes—it is being implemented in accordance with the provisions of the pilot program in the form of three reference levels.
	Were the current pilot program and the previous model consistent with the assumptions?	Yes—both the current pilot program and the earlier model were consistent with the assumptions regarding the provision of basic psychiatric care.	Yes—it represents a transition towards a community-based model.

Table 1 presents the comparative analysis of the previous and current models of child and adolescent mental health care, revealing notable differences in usefulness, effectiveness, and adequacy. In terms of usefulness, the previous model was hindered by prolonged waiting times and the absence of preventive measures, while the new model incorporates a preventive and early intervention approach, partially improving access and service comprehensiveness despite persistent waiting lists.

Regarding effectiveness, the previous model lacked clearly defined operational objectives and priority areas, whereas the current model is in an active implementation phase, expanding its network of facilities across all reference levels. Nevertheless, implementation has exceeded the initial timeframe, and budget constraints remain a significant barrier. The old model operated without standardized quality indicators, in contrast to the new model, which applies uniform organizational standards.

In the domain of adequacy, the earlier system struggled with high incidence rates and insufficient resources, while the current model addresses these challenges through planned comprehensive actions that integrate prevention, hospitalization, and community-based interventions. Both models were consistent with their initial assumptions; however, the new model represents a shift toward a community-based approach.

## 4. Discussion

Based on a review of the literature in the Google Scholar, PubMed, Web of Science, and Scopus databases, using keywords such as “CAMHS,” “Child and adolescent,” “mental health,” and “healthcare,” no comparable work to the above was found. Additionally, to deepen and complement the review, the analysis was extended to include a comparison of Poland’s situation with that of other member states of the Organisation for Economic Co-operation and Development (OECD). The OECD, currently comprising 38 highly developed and democratic countries, plays a key role in identifying, evaluating, and promoting exemplary policy solutions in the areas of economy, health care, and social policy. Its mission is not only to formulate evidence-based recommendations, but also to support member countries in achieving the highest possible levels of economic well-being, social cohesion, and sustainable development [28].

The presented table, prepared on the basis of OECD data from 2018 to 2022, compares Poland with other OECD countries in terms of the number of psychiatrists per 1000 inhabitants in each country’s population. It should be noted that there is no internationally recognized indicator that exclusively measures the number of specialists holding the title of child and adolescent psychiatrist per the population of children in individual countries. A potential avenue for future research is the examination of the hypothesis that in many countries, systemic solutions are applied based on similar diagnostic, therapeutic, and organizational frameworks. However, it must be emphasized that significant differences also exist—among others, in financing models, institutional structures, and the degree of decentralization of the health care system. Taking these diverse conditions into account may be crucial when interpreting the results and adapting foreign solutions to the Polish context.

At the same time, the limitations of such analyses should be recognized, arising from differences in data quality and completeness, as well as from the varied socio-economic and geographical contexts of OECD member states [29]. Norway can serve as an illustrative example, often cited as a model in health care systems. However, its system is highly specific and cannot be directly compared to that of Poland—primarily due to geographical factors and its small population. These factors have necessitated, among other measures, the development of telemedicine, enabling Norwegian solutions to have potential applicability in other countries through innovative approaches. Moreover, Norway is among the European countries with the highest levels of health care funding. Combined with its unconventional approach, this makes Norway a frequent point of reference in shaping comprehensive health care systems—including in the field of child and adolescent psychiatry [30].

The countries selected for comparison were chosen based on the diversity of their health care systems (the market-based model in the United States, the Beveridge model in the United Kingdom) and the noted changes in mental health care systems, with examples of implementing the community-centered model being Italy for adults and Norway for the entire population.

In the important study conducted by Signorin et al., selected aspects of the organizational architecture supporting child and adolescent mental health in Europe (28 European Union countries) are described. Among the identified factors were, for example, the number of beds in inpatient units—reaching a record high of 8400 in Germany. The study also highlighted numerous challenges and data gaps, as comprehensive datasets were obtained from fewer than 50% of the countries. Furthermore, the authors emphasized the urgent need for systemic reforms, particularly in larger countries, where issues were more pronounced due to factors such as a higher patient population, variability in service availability, and differences in provider types (public, private, or non-governmental organizations) [31].

Table 2 below, illustrating the number of child and adolescent psychiatrists per 1000 inhabitants, demonstrates that this figure has remained relatively stable over time. It is important to note that in selected countries—such as Australia, Colombia, the Czech Republic, and others marked with the letter “E”—the values presented are estimates. The highest numbers of psychiatrists per capita are observed in Switzerland, Germany, the Netherlands, Lithuania, and Greece, whereas the lowest are reported in Mexico, Turkey, and South Korea.

One of the similar studies discusses one of the most well-known reforms of the mental health system (for adults) in Italy, which serves as a flagship example of the implementation of the community-based psychiatry model [33]. Both studies emphasize the need for implementing comprehensive care in the community as quickly as possible. A similar approach is seen in Poland’s reform of adult mental health care [34]. Which assumes a multifaceted and rapid access to care, from the moment of referral through the development of a diagnostic–therapeutic plan within 72 h, to the application of treatment in collaboration with the individual in crisis and their family. Given the growing scope of the reform, in areas covered by it, there is a good chance of transitioning from child and adolescent psychiatry to adult psychiatry for those with chronic mental disorders, which is very difficult in areas where the reform is absent. This often leads to an interruption of treatment, despite the fact that there are nearly eight times more adult psychiatrists (4860) than child and adolescent psychiatrists, with the psychiatrist serving as the gatekeeper to further care.

Although there is still a high demand for access to support, new facilities have been established under the reform to provide this assistance. However, there are threats that may affect, and in some cases already are affecting, the operation of entities at all reference levels. These are very similar to the issues faced outside the pilot program—funding and staff availability. Another factor threatening the availability of services, the restriction of admissions, or the temporary closure of facilities is the insufficient number of places relative to the needs. In inpatient wards, priority is given to those in life-threatening situations, either to themselves or others [2]. However, due to the lack of beds, patients sometimes have to be treated in another province or even in a pediatric ward, often unrelated to mental health care. Given that many individuals in this age group are experiencing chronic crises and the current limitations of available interventions in such urgent circumstances, finding an optimal solution is challenging. This issue is the subject of much discussion regarding the organization of child and adolescent mental health care.

As the challenges related to supporting children and adolescents’ mental health are international in nature, numerous action plans are being developed by major organizations that bring together various countries, both on national and international scales.

The organization of child and adolescent mental health care in the United States is multifaceted and dynamically evolving, with a focus on various intervention models and access to appropriate care. However, the system’s shortcomings are also emphasized. On the one hand, there are mental health protection laws, strategies, and a progressive transformation, similar to Poland. On the other hand, there are issues such as inadequate access to care, underfunding, the need for new treatment methods based on scientific research, ensuring preventive actions, and continuity of care [35,36].

Norway is an exemplary country in terms of mental health protection. Due to its mountainous terrain and other community challenges, it has developed a model system for e-health and telemedicine. Moreover, Norway has already actively implemented or is in the process of implementing key aspects of many national and international programs. These include mental health promotion and prevention, easy and quick access to specialists, and crisis care [37].

It also confirms the thesis about the lack of sufficient research and publications on child and adolescent mental health, the organization of mental health care, and data flow on this topic. It was also highlighted that the system should not only operate in a medical facility but should also be implemented in the community—school, leisure settings, and discussions with parents. There should be no hesitation in utilizing other supportive tools—telemedicine, support groups, and, as much as possible, flexible care [14,38]. Additionally, prevention is emphasized for several reasons. First, it is more cost-effective and helps detect issues at an early stage. It also helps to prevent chronic crises and reduce the wait for support. It also supports and educates individuals in remission or post-crisis. It is important that the growing numbers of these strategies are not only declared but also summarized and analyzed. It is also worth noting that modern models of care must recognize that mental health support should not only focus on medical and para-medical aspects. The need to truly help individuals and their families should not be forgotten, meaning the root of the problem must be addressed with an interdisciplinary approach. Individuals in crisis may face financial issues, a lack of access to work, legal problems, or conflicts in their immediate environment. Addressing these areas can significantly aid in resolving the crisis and improving the condition of the person in need. This issue also affects children and adolescents, both directly and indirectly (through family problems). Systematically implementing mental health education is crucial. Currently, this is carried out through territorial initiatives or the efforts of non-governmental organizations. Due to its impact on promoting mental well-being, raising awareness of concerning signs, and preventing stigmatization, education for both children and parents would be ideal. Some adults hold negative views on the concept of mental health and mental disorders, which can affect, for example, whether they give consent for their child’s treatment—an essential element of access to help. Complementing the aspects of the reformed mental health support system for children and adolescents provides a great opportunity for comprehensive care, prevention, and improving mental well-being in children and adolescents.

A significant and underappreciated form of support comes from non-governmental organizations that strive to offer preventive measures, early diagnosis, and, if possible, even treatment. Currently, some local centers run by non-governmental organizations operate similar day-care facilities. However, they function locally, and participation in activities requires the consent of a guardian for those under 18. These initiatives are funded through grants for tasks related to providing mental health support, voluntary donations, and tax deductions (PIT), as well as contracts with the National Health Fund for selected psychotherapy or psychiatric services. However, several limitations exist—the most notable being a lack of information about the services, financial constraints, and the requirement of parental consent for minors. The availability of services is also limited to specific areas, such as a city. Nevertheless, in areas where the “old system” still functions, these centers provide significant support, both in regions covered by the pilot program and in those where the needs exceed the available resources. They partially address problems and provide preventive actions to protect the local community served by the program or facility.

## 5. Conclusions

The organization of mental health care in Poland in the first half of the second decade of the 21st century is very slowly shifting towards community-based psychiatry. Both the awareness and organization of mental health support for children and adolescents remain focused on a medicalized model, although recent initiatives are slowly beginning to change this. Thanks to the efforts of psychiatric associations, some policy-makers, and non-governmental organizations in particular, help is now extending beyond the medical sphere, with more involvement from educators and psychologists in the early support of children. Psychological support centers and day-care facilities are being opened by public order organizations. Local educational and support programs are also being implemented. Despite these actions, due to delayed interventions in the past and limited capacity to support such a large number of children in crisis and difficult situations, there is a risk of delaying the delivery of appropriate help, which could lead to the transformation of a crisis into a chronic disorder requiring more interventionist and long-term actions. Moreover, the issue of access to care affects not only Poland but also many other countries, so the actions being taken must be urgently implemented [39]. It is important that these actions help to relieve psychiatrists, on whom the system heavily relies, and whose impact has not been sufficiently visible with the slow rollout of the pilot program in facilities under the “old system,” especially when many children reach help in an advanced crisis. It would also be valuable to adopt Norwegian supportive measures, which focus on developing mental health promotion, telemedicine, and pilot programs that are being consistently implemented. Thanks to the reform, there is a chance to improve the situation regarding preventive crisis responses, but it is also necessary to expand the infrastructure of the reference levels II and III due to the number of children requiring psychiatric support. Introducing preventive measures and health promotion activities closer to students, for example, in schools, would also be beneficial to respond to potential crises as early as possible.

Current analyses reveal significant gaps in data regarding the number of child and adolescent psychiatrists per population of children in individual countries. A review of the available literature highlights a substantial shortage of studies on outpatient mental health care indicators for children and adolescents, particularly in a cross-country comparative context. Data published in international reports, such as the WHO Mental Health Atlas [40], only include selected indicators, with many measures remaining unpublished or inaccessible to researchers. This limitation restricts the ability to conduct in-depth epidemiological analyses and to compare the effectiveness of different care models in diverse geographical and socio-economic contexts. Further research is needed, encompassing both quantitative and qualitative aspects of outpatient mental health services, with particular emphasis on accessibility, quality, and the effectiveness of interventions.

Moreover, there is a notable deficit of analyses assessing the number of child and adolescent psychiatry specialists in relation to the pediatric population across individual countries. This highlights the need for in-depth research in this area or the development of dedicated reports by international organizations. Such standardized and comparable data would enable the monitoring of workforce availability at a global level and the identification of regions requiring priority remedial actions.

From a health policy perspective, emphasis should be placed on strengthening community-based approaches and reducing the overmedicalization of mental health support for children and adolescents. Key priorities include increasing funding for child and adolescent psychiatry, supporting the education and training of professionals working with this population, and ensuring that mental health policy is driven by evidence-based needs rather than temporary political agendas.

At the European level, policy milestones should include the development of a unified EU mental health policy for children, accompanied by a dedicated directive for member states. Such legislation should establish minimum budgetary allocations—or at least recommended percentages—specifically for child and adolescent mental health, thereby ensuring sustained investment and harmonized standards across countries.

## Figures and Tables

**Table 2:** Psychiatrists per 1000 inhabitants in OECD countries [32].

	Time Period				
Reference Area	2018	2019	2020	2021	2022
Mexico	0.01.	0.01	0.01	0.01	0.01
Colombia	E 0.02	E 0.02	E 0.02		
Türkiye	0.05	0.06	0.06	0.07	0.07
Korea	0.08	0.08	0.08	0.09	0.09
Chile	0.1	0.1	0.11	0.11	0.12
Poland	0.09	0.12	0.12	0.13	0.13
Spain	0.11	0.12	0.12	0.13	0.13
Japan	0.13		0.13		0.13
Hungary	0.15	0.15	0.14	0.14	0.15
United States	0.14	0.14	0.14	0.14	0.15
Portugal	0.13	0.13	0.14	0.15	0.15
Latvia	0.16	0.16	0.15	0.15	0.15
Czechia		E 0.16	E 0.15	E 0.17	E 0.17
Israel	0.16	0.16	0.16	0.16	0.17
Slovenia	0.15	0.15	0.16	0.16	0.17
Belgium	0.17	0.17	0.17	0.17	0.17
Canada	0.17	0.17	0.17	0.18	0.18
Finland	0.18	0.19	0.18	0.18	
Italy	0.17	0.17	0.18	0.19	0.2
Australia	E 0.17	E 0.18	E 0.18	E 0.19	E 0.19
Denmark	0.19	0.19	0.19	0.19	
United Kingdom	0.18	0.18	0.19	0.2	E 0.2
Estonia	0.19	0.2	0.2	0.19	0.2
Iceland	0.24	0.21	0.2	0.2	0.21
New Zealand	E 0.19	E 0.19	E 0.2	E 0.2	E 0.21
Ireland	0.17	0.19	0.21	0.19	0.22
Austria		0.2	0.21	0.22	0.22
Sweden	0.23	0.22	0.22	0.22	
France	0.23	0.23	0.23	0.23	0.23
Norway	0.23	0.23	0.23	0.23	0.24
Greece	0.26	0.26	0.24	0.25	0.26
Lithuania	0.23	0.23	0.25	0.25	0.25
Netherlands	0.24	0.24	0.25	0.25	0.25
Germany	0.28	0.28	0.28	0.29	0.28
Switzerland	0.52	0.52	0.53	0.53	0.53

## Data Availability

Data is contained within the article.
